# Evolutionary Implications of a Peroxidase with High Affinity for Cinnamyl Alcohols from *Physcomitrium patens*, a Non-Vascular Plant

**DOI:** 10.3390/plants10071476

**Published:** 2021-07-19

**Authors:** Teresa Martínez-Cortés, Federico Pomar, Esther Novo-Uzal

**Affiliations:** 1 Grupo de Investigación en Biología Evolutiva, Centro de Investigaciones Científicas Avanzadas, Universidade da Coruña, 15071 A Coruña, Spain; teresa.martinez.cortes@gmail.com (T.M.-C.); federico.pomar@udc.es (F.P.); 2 Instituto Gulbenkian de Ciência, 2780-156 Oeiras, Portugal

**Keywords:** *Physcomitrella*, hydroxycinnamyl alcohols, plant evolution, peroxidase, abiotic stress

## Abstract

*Physcomitrium (Physcomitrella) patens* is a bryophyte highly tolerant to different stresses, allowing survival when water supply is a limiting factor. This moss lacks a true vascular system, but it has evolved a primitive water-conducting system that contains lignin-like polyphenols. By means of a three-step protocol, including ammonium sulfate precipitation, adsorption chromatography on phenyl Sepharose and cationic exchange chromatography on SP Sepharose, we were able to purify and further characterize a novel class III peroxidase, PpaPrx19, upregulated upon salt and H_2_O_2_ treatments. This peroxidase, of a strongly basic nature, shows surprising homology to angiosperm peroxidases related to lignification, despite the lack of true lignins in *P. patens* cell walls. Moreover, PpaPrx19 shows catalytic and kinetic properties typical of angiosperm peroxidases involved in oxidation of monolignols, being able to efficiently use hydroxycinnamyl alcohols as substrates. Our results pinpoint the presence in *P. patens* of peroxidases that fulfill the requirements to be involved in the last step of lignin biosynthesis, predating the appearance of true lignin.

## 1. Introduction

Land colonization by plants and their subsequent diversification was one of the most important events in the history of life. Terrestrialization forced plants to cope with new stresses absent in the aquatic medium, such as UV light and limited water supply. To avoid water loss, plants developed different strategies to accumulate water in their tissues, to supply it or to minimize its loss. These first land plants, such as mosses, were poikilohydric, whose water potential was equilibrated with surrounding water sources [1]. Likewise, the first evolutionary radiation among land plants is related to the diversification of tracheids, which appeared in vascular plants (tracheophytes), about 450 million years ago and they have been defined as single-celled conduits with lignin in their cell wall [2]. Lignin is a polyphenolic polymer that confers structural support and flexural stiffness to the aerial part of the plant and provides water impermeability, including resistance against tensile forces of water columns. Lignins are mainly formed from the oxidative coupling of three *p*-hydroxycinnamyl alcohols: *p*-coumaryl, coniferyl and sinapyl alcohols (monolignols). The cross-coupling reaction of monolignol radicals produces a hydrophobic heteropolymer composed of *p*-hydroxyphenyl (H), guaiacyl (G) and syringyl (S) units [3].

Although lignin has traditionally been linked to vascular plants, polyphenols and lignin-like compounds have been found in species without a true vascular system, such as charophycean green algae [4] and bryophytes [5,6]. Lignin-like compounds are polyphenolic polymers usually detectable with typical methods of lignin determination, such as acetyl bromide or nitrobenzene oxidation, but unlike lignin, they lack β-*O*-4 bonds and aryl-glycerol-β-aryl ether structures. The composition is very variable and in many cases unknown but not related to the three *p*-hydroxycinnamyl alcohols that are considered markers for lignins. This finding implies that at least part of the phenylpropanoid pathway that eventually led to lignin biosynthesis was present in algae, and the presence of lignin in tracheids may only have involved the expression of those genes in a different type of cell [7].

The last step of lignin biosynthesis, the oxidation of monolignols, is driven by laccases [8] and peroxidases [9]. Secretory plant peroxidases (class III Prx) are heme-containing glycoproteins that oxidize diverse substrates using hydrogen peroxide as an electron donor. Peroxidases are usually rich in isoenzymes, generated from post-transcriptional and post-translational modifications [10], with expression patterns usually dependent on development and stress conditions, which make it difficult to assign specific functions to individual peroxidase isoenzymes. Nonetheless, diverse responses to a plethora of stresses or growth conditions have been reported, especially in *Arabidopsis*, indicating specific functions for the different isoforms [9,11]. 

*Physcomitrium (Physcomitrella) patens* is a bryophyte used as a model organism for evolutionary developmental biology and non-vascular plant studies. *P. patens* shows high tolerance to different environmental cues, such as drought and osmotic and saline stresses, which allows survival in periods when water supply is a limiting factor [12]. RedoxiBase reports 53 class III peroxidases and four pseudogenes in the *P. patens* genome. However, information about *P. patens* peroxidase functions is scarce. The best characterized peroxidase is Prx34 (PpaPrx13 according to RedoxiBase nomenclature), which was reported to play a role upon fungal attack and catalyze ROS production [13,14]. 

In this paper, we report the purification of one *P. patens* peroxidase, upregulated upon salt and oxidative stresses. This enzyme was further purified and characterized, showing homology to angiosperm peroxidases involved in lignification, and with a catalytic efficiency against coniferyl alcohol, a precursor of lignin, of the same order as angiosperm lignification-related peroxidases, despite the fact that *P. patens* does not contain lignin in its cell walls. 

## 2. Results

### 2.1. Abiotic Stress Strongly Modulates the Expression of a Basic Peroxidase in P. patens

**Figure 1:**
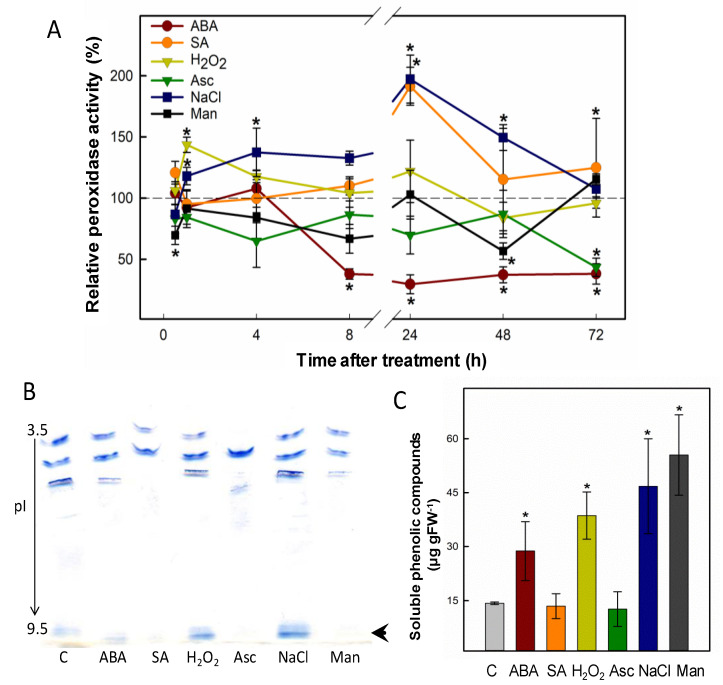
Salt and oxidative stress upregulate the expression of a basic peroxidase from *P. patens*. (**A**) Time-course determination of peroxidase activity extracted from *P. patens* gametophores that were subjected to different stress treatments: H_2_O_2_ with or without ascorbic acid (Asc), salt, mannitol (Man), abscisic acid (ABA) and salicylic acid (SA). TMB was used as substrate and peroxidase activity was normalized to control conditions at each time point (shown as a dashed line). (**B**) Effect at 24 h of different treatments on peroxidase isoenzyme pattern revealed by IEF stained with 4-MN. C, control; pI, isoelectric point. (**C**) Effect of stress treatments at 96 h on soluble phenolic content in *P. patens* cultures. Data presented are average values ± SD of *n* = 3 experiments. Statistical analysis was carried out with Duncan’s test, asterisks represent statistical differences from control (*p* < 0.05).

*P. patens* is a moss that is highly resistant to abiotic stress, compared to other model plants such as *Arabidopsis thaliana*, and is especially tolerant to desiccation [15], in line with its phylogenetic position as a moss, and whose ancestors were early colonists of land around 500 million years ago. This bryophyte is thus a useful tool to study responses to abiotic stress. Peroxidases are enzymes known to change their expression pattern in response to different types of stress [16,17]. Here, we selected different abiotic stresses and monitored in a time course both peroxidase activity and isoform pattern from protein extracts of *P. patens* gametophores grown in liquid medium. The results (Figure 1A) show that H_2_O_2_ caused an early increase in peroxidase activity, peaking 1h after treatment. The addition of ascorbic acid, a known H_2_O_2_ scavenger, returned peroxidase activity to control levels. NaCl and salicylic acid (SA) also enhanced peroxidase activity 24 h after treatment, going back to control levels afterwards. Mannitol slightly changed peroxidase activity throughout the treatment. Although both osmotic and salt stress can be abscisic acid (ABA) dependent, *P. patens* peroxidase activity in response to ABA treatment did not mirror mannitol or salt stress responses, but strongly decreased from 8 h after the hormone addition. Moreover, SA and mannitol led to the disappearance of a strongly basic peroxidase isoform, which was instead induced by H_2_O_2_ and NaCl (Figure 1B, arrow). We quantified free phenolics as a proxy to measure the stress caused by the different treatments. H_2_O_2_, salt and mannitol treatments significantly enhanced the amount of total phenols in *P. patens* gametophores (Figure 1C). Based on these results, we pursued the purification and further characterization of a strongly basic peroxidase that was induced by H_2_O_2_ and salt, two major stresses faced by the first plants that colonized land. 

### 2.2. PpaPrx19 Is a 36 kDa Basic Peroxidase

**Figure 2:**
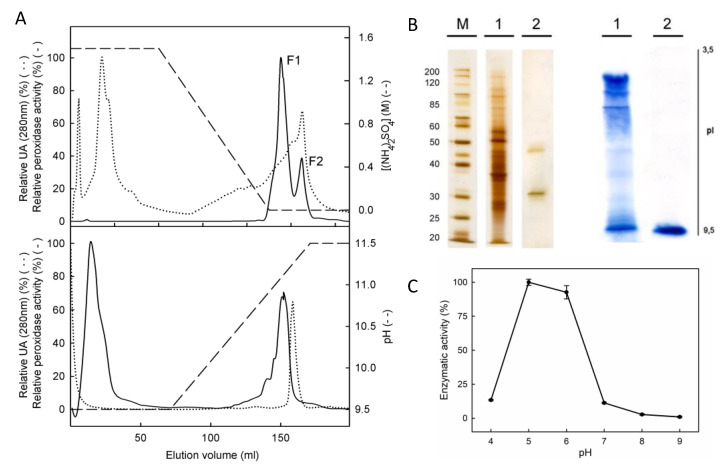
Purified PpaPrx19 is a strongly basic peroxidase. (**A**) Purification process of PpaPrx19, including adsorption chromatography on phenyl Sepharose (upper panel, peak F2) and cationic exchange chromatography on SP Sepharose (lower panel). Profiles of peroxidase activity and protein are denoted either by a continuous or by a dotted line, respectively. (**B**) Protein fingerprint in SDS-PAGE (left) and peroxidase isoenzyme pattern in IEF (right) of the crude extract (1) and the purified peroxidase (2) SDS-PAGE and IEF were revealed using the silver staining method and 4-MN in the presence of H_2_O_2_, respectively. (**C**) Dependence on pH of the purified PpaPrx19 activity. Data presented are average values ± SD of *n* = 3 experiments.

We extracted total protein from *P. patens* gametophores grown in control conditions and we then followed a three-step protocol for purification, including ammonium sulfate precipitation, hydrophobic chromatography on phenyl Sepharose and cationic chromatography on SP Sepharose. A fractionated precipitation with ammonium sulfate did not allow the partial purification of the peroxidases of interest, which led us to consider just one fraction, precipitating the proteins with 95% (NH_4_)_2_SO_4_. The fraction was pooled into phenyl Sepharose chromatography, obtaining two major fractions of peroxidases (Figure 2A). The first eluted fraction (F1) contained only acidic peroxidases, while F2 contained both neutral and basic peroxidases and therefore was selected to continue the purification process (Figure 2). After this step, the specific activity for F2 reached 266 nkat mg^−1^ protein (Table 1). The F2 was then loaded into a cationic exchange chromatography, and the peroxidase bound to SP Sepharose matrix was eluted with a linear gradient of 9.5–11.5 pH. Neutral peroxidases were not retained in the matrix and only one peak of peroxidase activity was eluted, at a pH of 10.9 (Figure 2A). The fraction arising from cationic chromatography migrated as two different bands of 36 and 46 kDa in SDS-PAGE electrophoresis, but an IEF showed only one peroxidase with the pI value determined to be 10.04 (Figure 2B). The peptide mass fingerprinting of the two resultant proteins enabled us to detect that the protein of 46 kDa corresponded to a lipase (accession number XP_001755452). The 36 kDa protein was identified as a predicted protein (access number XP_001781554) which corresponded to PpaPrx19 (according to RedoxiBase nomenclature).

We confirmed by RT-qPCR that *PpaPrx19* was strongly induced after a treatment with NaCl and that gene expression was modulated in response to other stresses such as hydrogen peroxide and mannitol (Figure S1). 

We also evaluated the dependence on pH of PpaPrx19 enzymatic activity, using a different pH (4.0 to 9.0) in the reaction mixture. The purified peroxidase showed the highest activity at pH 5.0, but it rapidly decreased at pH > 6 and showed no activity at pH above 7.0 (Figure 2C). These results do not differ from other peroxidases purified from different sources, with the optimum pH between 4.5 and 6.5 [18,19,20]. pH is critical for peroxidase activity because pH values outside the optimum prevent the heme from binding to the active site of the enzyme [21].

### 2.3. PpaPrx19 Is Homologous to Peroxidases with a Role in Lignification

PpaPrx19 is 332 amino acids long, including a 26 aa N-terminal signal peptide, and it is targeted to the secretory pathway according to analysis with SIGNALP [22] and TARGETP [23] programs. The exon–intron pattern of PpaPrx19 is the second most abundant for *P. patens* and classic for class III peroxidases, consisting in three exons and two introns [13]. In a BLAST search, PpaPrx19 showed the highest identity at the protein level with two other *P. patens* peroxidases (PpaPrx18 and PpaPrx09) and the moss *Tortula ruralis* (Table 2). The rest of the listed peroxidases belong to gymnosperms and angiosperms, and show identity values below 50%, emphasizing the evolutionary distance among them and pointing out the unique characteristics of this peroxidase, at the amino acid level. 

Given these low identity values, we blasted PpaPrx19 against *Arabidopsis* peroxidases, in order to infer a putative function. Most of the peroxidases with the highest identity level have a reported role in lignification (Table 3). This was a surprising result, given that *P. patens* has an internal water-conducting system constituted by hydroids and living cells with thick walls [24] containing pre-lignin and lignin-like polyphenols but no true lignin (defined as the polymerized compounds found in vascular plants) has been described [5,6]. 

### 2.4. PpaPrx19 Shows High Affinity for Cinnamyl Alcohols

The homology that PpaPrx19 shows with peroxidases reportedly involved in lignification, together with the reported presence of lignin-like polyphenols in *P. patens*, led us to characterize this peroxidase based on its preferred substrates, using different well-known peroxidase substrates, including natural precursors of lignin monomers (Table 4). Ascorbic acid is a typical substrate for class I (ascorbate) peroxidases but is poorly oxidized by class III secretory peroxidases [33]. The oxidation of NADH by peroxidases has been associated with cell wall loosening [34]. IAA is an in vitro peroxidase substrate and it has been reported to be catalyzed in vivo in relation to cell growth [35]. Hydroxycinnamic acids such as ferulic acid can be incorporated into suberin [36] and ferulate can also lead to cross-linking of the cell wall [37]. Coniferyl and sinapyl alcohols are polymerized by apoplastic peroxidases to form lignin [38]. Results showed that PpaPrx19 is able to use each assayed substrate, except ascorbic acid and NADH, although IAA was a poor substrate for PpaPrx19. This peroxidase is able to oxidize ferulic acid (0.53 ± 0.07 nkat μg^−1^ protein) and sinapyl alcohol (0.08 ± 0.01 nkat μg^−1^ protein) but the highest activity is shown using coniferyl alcohol as a substrate (1.46 ± 0.13 nkat μg^−1^ protein).

Trying to decipher a putative role in cinnamyl alcohol oxidation, we determined the catalytic parameters of PpaPrx19 for coniferyl and sinapyl alcohols. To calculate the kinetic constants, hydrogen peroxide was used at saturation levels (0.5 mM). The *K*_M_ values were calculated according to Lineweaver–Burk equations. For PpaPrx19, apparent *K*_M_ values were similar for both alcohols (16.7 μM for coniferyl alcohol and 20.8 μM for sinapyl alcohol). However, *K*_cat_ is much higher for coniferyl alcohol, rendering a higher catalytic efficiency (*K*_cat_/*K*_M_), making coniferyl alcohol the best substrate (Table 5). 

**Figure 3:**
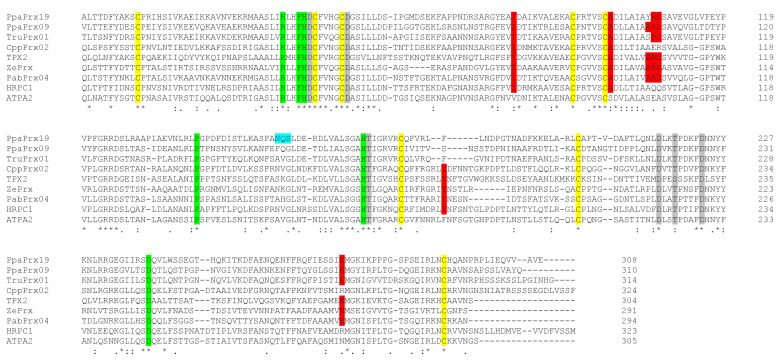
PpaPrx19 shows most of residues characteristic of syringyl peroxidases. Amino acid alignment of mature peroxidase sequences, including those purified in this study (PpaPrx19), *Arabidopsis thaliana* ATP A2 (CAA68212), horseradish peroxidase HRPC1 (AAA33377), *Picea abies* PabPrx04 (CAH10839), *Zinnia elegans* ZePrx (CAI54302), *Solanum lycopersicum* TPX2 (AAA65636) and the three peroxidases that show the highest identity to PpaPrx19 (PpaPrx09, TruPrx01 and CppPrx02). Conserved residues important for catalytic mechanisms are shaded in green, the calcium-binding sites are shaded in gray, the S-S bridge-forming cysteines are shaded in yellow, putative *N*-glycosylation site of PpaPrx19 is shaded in blue and structural determinants of syringyl peroxidases are shaded in red. Consensus symbols: ‘*’ indicates fully conserved residues, ‘:’ indicates conserved substitutions and ‘.’ indicates semiconserved residue substitutions.

With these extraordinary biochemical characteristics in mind, we searched for structural determinants that define a particular type of isoenzyme, the syringyl peroxidases [39]. We aligned PpaPrx19 with peroxidases with experimental capacity for oxidizing sinapyl alcohol, including ZePrx (the paradigmatic syringyl peroxidase); ATP A2 and HRP which are unable to oxidize sinapyl alcohol; and the three peroxidases that show the highest identity to PpaPrx19, as shown in Table 2. PpaPrx19 not only contains conserved residues important for catalytic mechanisms and the amino acids required for coordination of two Ca^2+^ ions (Figure 3), but it also presents most of the structural determinants of syringyl peroxidases (marked in red in Figure 3), which suggests that this peroxidase has no structural restrictions to oxidizing sinapyl alcohol [39]. As a matter of fact, the PpaPrx19 catalytic properties suggest this peroxidase shows a low *K*_M_ for sinapyl alcohol and its ability to oxidize this substrate in vitro (Table 5). 

### 2.5. PpaPrx19 Associates with Lignin Biosynthesis Enzymes and Cell Wall-Related Proteins

Finally, we searched for protein associations by means of STRING (string-db.org). This program provides a network of predicted associations for a particular group of proteins based on high-throughput experimental data, literature and database mining [40]. In the case of PpaPrx19, it is located in the center of a network comprising 10 proteins (Figure S2), which are listed as having unknown functions in the poorly annotated *P. patens* genome. We then searched, for each predicted *P. patens* protein, its closest homolog in *Arabidopsis*. The results are shown in Table 6. The proteins with the highest score are three cinnamyl alcohol dehydrogenases (CADs) and one O-methyl-transferase (OMT). CAD participates in the lignin biosynthetic pathway, catalyzing the conversion of cinnamyl aldehydes into their corresponding alcohols. Unfortunately, the CAD proteins identified by STRING have not been characterized biochemically, although they are known to be expressed in lignifying tissues [41]. The associated OMT has been reported to have high affinity (in the µM range) for a plethora of phenylpropanoids, such as coniferyl alcohol and aldehyde, as well as quercetin [42]. 

We also performed an analysis in Phytozome (https://phytozome.jgi.doe.gov/pz/portal.html; accessed on 8 July 2021) and searched for coexpression patterns with PpaPrx19. The list (Table S1) comprises phenylalanine ammonia-lyase (PAL), the first enzyme of phenylpropanoid metabolism, which includes the branch that leads to lignin formation [3]. Other enzyme-encoding genes are also coexpressed with PpaPrx19, such as β-1,3-glucanase-related and exostosin heparin sulfate glycosyltransferase-related, both associated with remodeling of the cell wall [43]. Moreover, the WRKY transcription factors have been reported to be involved in the regulation of lignin deposition [44]. These associations support the putative involvement of PpaPrx19 in the formation of lignin or lignin-like compounds. 

## 3. Discussion

The cell wall is characteristic of all plant cells, although its composition varies depending on the cell type, the lineage and environmental conditions. Therefore, the plant cell wall is essential for cell development and in responses to stress, being able to plastically adapt to the cell’s needs. Several innovations that arose during plant evolution, such as lignin and suberin, help to promote this plasticity. Lignin is thought to have emerged with vascular plants 450 million years ago, but lignin-like or pre-lignin compounds have been detected in bryophytes and algae [5,45]. The last step of lignin biosynthesis, the oxidation of hydroxycinnamyl alcohols, is catalyzed by class III peroxidases. In this work, we report the purification of a *P. patens* peroxidase, PpaPx19, with the ability to oxidize hydroxycinnamyl alcohols (Table 4 and Table 5). This characteristic is surprising not just because *P. patens* does not lignify, but also because of the atypical kinetic properties shown by this peroxidase. In our experiments, PpaPrx19 showed *K*_M_ values for hydroxycinnamyl alcohols similar to other peroxidases involved in lignification, from flowering and non-flowering plants. While PpaPrx19 showed *K*_M_ values of 16.7 and 20.8 μM for coniferyl and sinapyl alcohols, respectively (Table 5), the gymnosperm *Picea abies* contains two basic peroxidases involved in lignification with reported *K*_M_ values for coniferyl alcohol of 16.7 and 23.2 μM [46]. In angiosperms, *K*_M_ values have been reported for zinnia (83 µM for coniferyl alcohol) and tomato (11.4 µM for syringaldazine, a chemical analog of sinapyl alcohol) [47,48]. 

The use of catalytic efficiency (*K_cat_/K*_M_) is preferable in order to compare diverse enzymes and substrates, although very few peroxidase reports calculate this parameter to evaluate enzyme kinetics. In the lycophyte *Selaginella,* two basic peroxidases show values of 3.55 and 28.63 µM^−1^s^−1^ with coniferyl alcohol [20]. GbPrx09 from *Ginkgo biloba*, a gymnosperm, showed values of 4.91 µM^−1^s^−1^ for coniferyl alcohol [17]. In dicots, ZePrx from *Z. elegans* showed a *K_cat_/K*_M_ ratio for coniferyl alcohol of 1.20 µM^−1^s^−1^ [48] and TPX1 (from tomato) showed a *K_cat_/K*_M_ for syringaldazine of 1.50 μM^−1^s^−1^ [47]. In monocots, PviPRX9 from *Panicum virgatum* showed a *K_cat_/K*_M_ ratio for coniferyl alcohol of 1.60 µM^−1^s^−1^ [49].

The *K_cat_/K*_M_ value obtained for PpaPrx19 using coniferyl alcohol as a substrate is not only higher than for peroxidases involved in lignification [48], but also for other enzymes involved in phenylpropanoid metabolism, such as PAL (the first enzyme of the route, [50]), CCR (the first committed enzyme of lignin biosynthesis, [51]) and CAld5H, involved in last steps of lignin biosynthesis [52]. 

Moreover, our results indicate that PpaPrx19 is able to efficiently use sinapyl alcohol as a substrate. While coniferyl alcohol is easily oxidized by most peroxidases, the capacity of these enzymes to oxidize sinapyl alcohol is not such a common fact and defined a new subgroup named syringyl peroxidases [39]. PpaPrx19 has most of the structural determinants of this new subgroup [39] in the protein primary structure (Figure 3, shaded in red). These structural motifs determine the syringyl oxidase activity shown by peroxidases, but are absent in the two paradigmatic G peroxidases, ATP A2 and HRP A2 from *Arabidopsis* and horseradish, respectively. These structural motifs agree with the experimental capacity of PpaPrx19 of oxidizing sinapyl alcohol in vitro.

All these data strongly suggest that the peroxidase PpaPrx19 may have been involved in lignin biosynthesis, if such a pathway was present in *P. patens*, i.e., PpaPrx19 fulfills the kinetic and structural requirements to oxidize coniferyl alcohol. The presence of an enzyme involved in the biosynthetic route of a compound that appeared later in an evolutionary context is not surprising. Thermospermine emerges as one example of a metabolite typical of vascular plants recently described in non-vascular plants. The only reported function for thermospermine is the regulation of xylem cell maturation, which makes the function it may have in non-vascular plants unclear [53]. It is widely accepted that promiscuous enzymes with several putative substrates are more likely to be recruited to novel metabolic routes [54]. Therefore, a peroxidase with multiple substrates but with particular affinity for coniferyl alcohol would be a good candidate to participate in what eventually would constitute the pathway leading to lignin formation.

Several reports [55,56,57] indicate that the first appearance of the entire lignin biosynthesis pathway enzymes (excluding the pathway that leads to syringyl lignin formation), from the catalysis of phenylalanine to coniferyl alcohol formation, took place in mosses (*P. patens*), regardless the fact that *P. patens* does not accumulate lignin in the cell wall. Thus, the *P. patens* genome has all the genes necessary for the biosynthesis of lignin, and according to the results presented in this paper, at least some of the enzymes are expressed and functional, but the route does not take place and lignin is not polymerized. Nonetheless, a pre-lignin pathway has been recently suggested, revealing a role of caffeate units for the formation of the *P. patens* cuticle, coupled with ascorbate metabolism [6]. This finding suggests that the biosynthesis of lignin-like or pre-lignin compounds may not originate from the precursors described for canonical lignin and that the enzymes involved in its synthesis have broader specificity than the enzymes participating in true lignin from vascular plants, making lignin evolution an exciting field to explore.

PpaPrx19 may have a function that in vascular plants was later derived for involvement in lignification. This hypothesis is supported by its structural and kinetic homology to peroxidases with an already described role in lignin biosynthesis, such as ZePrx [39,48], and association with other enzymes of the lignin biosynthetic route. The appearance of a primitive water-conducting system, together with stomata and cuticle, were innovations developed by plants during the transition from water to land. Renault [6] already proved the existence of a pre-lignin pathway involved in the formation of the *P. patens* cuticle. Given all this, although the actual function of PpaPrx19 in *P. patens* physiology remains unclear and should be further studied, it is likely involved in the remodeling of the cell wall in response to environmental stress, based on this peroxidase ability of oxidizing phenolic compounds and its upregulation upon several conditions related to water deficiency, the paradigmatic stress for poikilohydric plants lacking a true vascular system.

## 4. Materials and Methods

### 4.1. Plant Material and Treatments

*P. patens* was provided by the Biotechnology Department of the Universidad Politécnica (Madrid, Spain) and cultured as described previously [58]. *P. patens* gametophores were maintained in solid medium under standard conditions in a growth chamber at 25 °C with a 16 h photoperiod. For stress treatments, *P. patens* gametophores were grown in liquid medium on a rotator shaker (130 rpm) and then transferred into medium supplemented with 5 mM H_2_O_2_ (with or without 10 mM ascorbic acid), 250 mM NaCl, 250 mM mannitol, 10 µM abscisic acid (ABA) and 1 mM salicylic acid (SA), respectively [59,60,61,62]. 

### 4.2. Protein Extraction and Precipitation

Gametophore samples (400 g) were homogenized in 50 mM Tris-HCl buffer (pH 7.5) containing 1 mM EDTA, 1 M KCl, and 0.05 g PVPP per g of tissue. The homogenate was filtered through nylon layers and centrifuged at 27,000× *g* for 30 min at 4 °C. The supernatant was dialyzed on cellulose membranes. After protein precipitation with 95% saturation ammonium sulfate, the precipitate was resuspended in 50 mM Tris-HCl pH 7.5 and dialyzed overnight against the same buffer. 

### 4.3. Purification of PpaPrx19

Purification of *P. patens* peroxidase 19 was performed in the AKTA System (GE Healthcare, Barcelona, Spain). The dialyzed sample was concentrated in Amicon^®^ Ultra (Merck Millipore, Barcelona, Spain) and dissolved in 1.5 M (NH_4_)_2_SO_4._ In the first step, hydrophobic chromatography, proteins were separated on a phenyl Sepharose^TM^ 6 Fast Flow (GE Healthcare, Barcelona, Spain) 31.5 × 1.0 cm gel bed column at a flow rate 1.0 mL·min^−1^, and fractions of 5.0 mL were recovered. The eluent chromatography program was as follows: from 0 to 160 min (100% A, 0% B), from 160 to 360 min (0% to 100% B) and from 360 to 515 min (100% B), where buffer A was 50 mM Tris-HCl (pH 7.5) containing 1.5 M (NH_4_)_2_SO_4_, and buffer B was 50 mM Tris-HCl (pH 7.5).

The second step involved ion-exchange chromatography on SP Sepharose Fast Flow (GE Healthcare). To do so, the peroxidase-enriched fractions obtained from the hydrophobic chromatography were dialyzed against 50 mM CAPS, (pH 9.5), and loaded on a 17.5 × 1.0 cm gel bed column equilibrated with 50 mM CAPS (pH 9.5), at a flow rate of 1.0 mL min^−1^. Fractions of 1.0 mL were recovered. The eluent chromatography program was as follows: from 0 to 70 min (100% A, 0% B), from 70 to 170 min (0–100% B) and from 170 to 330 min (100% B), where buffer A was 50 mM CAPS (pH 9.5), and buffer B was 50 mM CAPS (pH 11.5).

### 4.4. Peroxidase Activity Determination

Peroxidase activity was determined in a spectrophotometer at 25 °C in a reaction medium containing 50 mM sodium acetate buffer (pH 5.0) and 0.5 mM H_2_O_2_ with tetramethylbenzidine (TMB) 0.1 mg·mL^−1^ as substrate (ε_652_ = 39.0 mM^−1^·cm^−1^). 

Peroxidase activity was also calculated using different substrates: 100 µM coniferyl alcohol (ε_262_ = 9.75 mM^−1^·cm^−1^), 100 µM sinapyl alcohol (ε_271_ = 4.14 mM^−1^·cm^−1^), 50 µM ferulic acid (ε_310_ = 16.6 mM^−1^·cm^−1^), 200 µM indole-3-acetic acid (ε_261_ = 3.2 mM^−1^·cm^−1^), 1 mM ascorbic acid (ε_290_ = 2.8 mM^−1^·cm^−1^) and 150 µM NADH (ε_340_ = 6.22 mM^−1^·cm^−1^), as described elsewhere [48,63]. For measuring peroxidase activity with ascorbic acid, 50 mM phosphate buffer (pH 7.0) was used.

The pH-dependent enzymatic activity was assessed using 50 mM sodium acetate buffer for pH 4.0 to 6.0 and 50 mM Tris-HCl for pH 7.0 to 9.0, with TMB as substrate.

### 4.5. Kinetic Data Analysis

For determination of apparent *K_M_*, 5–100 μM (coniferyl alcohol) and 1–110 μM (sinapyl alcohol) concentrations of each substrate were used in 50 mM sodium acetate buffer pH 5.0. *K*_M_ values were determined from the Lineweaver–Burk equation, a linear transformation of the Michaelis–Menten equation. 

### 4.6. Electrophoretic Analysis 

Isoelectric focusing was performed on a Pharmacia Multiphor II system using Ampholine PAGplate (pH = 3.5–9.5) polyacrylamide gels according to the manufacturer’s instructions (GE Healthcare). Peroxidase isoenzymes were stained with 4-methoxy-α-naphthol (4-MN) in the presence of H_2_O_2_. 

SDS-PAGE was performed on 10 % (*w*/*v*) polyacrylamide gels using a MiniProtean^®^ 3 Cell electrophoresis kit (Bio-Rad Laboratories, Barcelona, Spain) and a pH 8.8 electrophoresis buffer composed of 192 mM glycine and 25 mM Tris containing 0.1% SDS. SDS-PAGE was performed at 200 V for 40–45 min at room temperature. Proteins were stained with a Plus One Silver Staining kit, according to the manufacturer’s instructions (GE Healthcare).

### 4.7. Phenolic Compound Extraction and Quantification

Gametophores grown in liquid culture were harvested 96 h after treatment, ground in liquid nitrogen and extracted for soluble phenolic content as previously described [17]. The quantitative determination of phenolics was performed using Folin–Ciocalteu reagent with ferulic acid as standard.

### 4.8. Molecular Weight MALDI-TOF/TOF

The purified peroxidase was analyzed in a MALDI-TOF/TOF instrument as previously described [20]. The search for peptide mass fingerprints and tandem MS spectra was performed in the NCBInr database without taxonomy restriction. Mascot scores for all protein identifications were higher than the accepted threshold for significance (at the *p* < 0.050 level, positive rate measured to be 0.047).

### 4.9. Sequence Data Analysis

The presence of signal peptides was predicted using TargetP (http://www.cbs.dtu.dk/services/TargetP/, accessed on 24 November 2020), Bacello (http://gpcr2.biocomp.unibo.it/bacello/index.htm, accessed on 24 November 2020) and TargetLoc (http://abi.inf.unituebingen.de/Services/MultiLoc/index_html, accessed on 24 November 2020). pI prediction was carried out using Compute pI/MW tool from ExPASy (http://web.expasy.org/compute_pi, accessed on 24 November 2020). A search for protein sequences homologies was performed using BLASTP from Redoxibase (http://www.peroxibase.toulouse.inra.fr, accessed on 4 February 2021) and protein alignments were carried out using Clustal Omega (https://www.ebi.ac.uk/Tools/msa/clustalo/, accessed on 4 February 2021). Search for *N*-glycosylation sites was performed using the NetNGlyc tool (http://www.cbs.dtu.dk/services/NetNGlyc, accessed on 25 November 2020).

### 4.10. Gene Expression Analysis by RT-qPCR 

Total RNA was isolated from gametophores grown in liquid medium, 24h after treatment, with Trizol (Invitrogen, Madrid, Spain) essentially as described in [64]. cDNA was synthesized from 200 ng of total RNA using the iScript™ cDNA Synthesis Kit (Bio Rad Laboratories, Barcelona, Spain). For RT-qPCR, the constitutively expressed *18S* gene was used as a reference gene (Fwd 5′-GGACCGATAGGTCTGGGTAA-3′ and Rev 5′-GCAATCCGAAAACTTCACCG-3′) and for *PpaPrx19* amplification, the primers Fwd 5′- CTCACCACTGACTTCTACGC-3′ and Rev 5′-TGGGATGCTGTCCAAGAGTA-3′ were used. The PCR reaction contained Bio-Rad 1x iQ SYBR Green Supermix, 0.3 µM primer mix and 2.5 µg of cDNA for a 50 µL final volume. The PCR program comprised a 1 min denaturation step at 94 °C followed by 40 cycles of amplification (94 °C for 30 s, 58 °C for 45 s and 72 °C for 1 min) and a final elongation step of 6 min at 72 °C. Bio-Rad Optical System Software 3.0 was used for data analysis, and relative expression values were calculated from the resulting Ct values [65]. 

### 4.11. Network Construction of PpaPrx19

The Search Tool for the Retrieval of Interacting Genes/Proteins database (STRING v11) was used to construct the network associated with PpaPrx19. The sequence of PpaPrx19 was loaded in the database and STRING generated a protein–protein interaction (PPI) network with proteins that have interactions with the target protein. Interactions were obtaining with medium confidence from curated databases and textmining. 

## Figures and Tables

**Table 1:** Purification of basic peroxidase PpaPrx19 from *P. patens*. Peroxidase activity was measured using TMB as substrate.

	Peroxidase Activity (nkat)	Specific Activity (nkat mg^−1^ Protein)	Purification Fold	Yield (%)
95% (NH_4_)_2_SO_4_ precipitation	698	28	1	100
Phenyl Sepharose chromatography	483	266	10	69
SP Sepharose chromatography	218	23,644	850	31

**Table 2:** Comparison of PpaPrx19 mature protein sequence with other class III peroxidases. The most similar peroxidase sequences based on BLASTP searches against the RedoxiBase database were used.

Prx Name	Species	Taxonomical Group	Identity (%)	E-Value
PpaPrx18	*Physcomitrium patens*	Bryophyte	66	3 × 10^−144^
TruPrx01	*Tortula ruralis* (star moss)	Bryophyte	65	3 × 10^−133^
PpaPrx09	*Physcomitrium patens*	Bryophyte	61	1 × 10^−124^
CppPrx02	*Citrus x Paradisi x Poncirus*	Angiosperm	49	3 × 10^−97^
PtaPrx102	*Pinus taeda* (loblolly pine)	Gymnosperm	50	8 × 10^−97^
CsPrx62	*Citrus sinensis*	Angiosperm	49	2 × 10^−96^
PabPrx05	*Picea abies* (Norway spruce)	Gymnosperm	49	6 × 10^−95^
TsPrx15	*Thellungiella salsuginea*	Angiosperm	47	1 × 10^−94^
BrPrx15-1	*Brassica rapa*	Angiosperm	48	6 × 10^−94^
PtaPrx28	*Pinus taeda* (loblolly pine)	Gymnosperm	46	9 × 10^−94^
PabPrx125	*Picea abies* (Norway spruce)	Gymnosperm	48	4 × 10^−93^
GbPrx04	*Ginkgo biloba*	Gymnosperm	49	6 × 10^−93^

**Table 3:** Reported function of *Arabidopsis* peroxidases which show highest homology to mature PpaPrx19 protein sequence after a BLAST search with Redoxibase.

Peroxidase Name	TAIR Gene ID	Identity (%)	E-Value	Function	Reference
AtPrx15	At2g18150	47	4 × 10^−93^	Lignification/abiotic stress	[25]
AtPrx49	At4g36430	47	6 × 10^−93^	Lignification	[26]
AtPrx53	At5g06720	46	1 × 10^−92^	Lignification	[27]
AtPrx14	At2g18140	48	2 × 10^−92^	Biotic stress	[28]
AtPrx22	At2g38380	47	5 × 10^−91^	Cold tolerance	[29]
AtPrx52	At5g05340	48	1 × 10^−89^	Lignification	[30]
AtPrx32	At3g32980	45	5 × 10^−88^	Cell elongation	[31]
AtPrx34	At3g49120	45	7 × 10^−88^	Oxidative burst	[32]

**Table 4:** Enzymatic activities (nkat μg^−1^ protein) of purified peroxidase PpaPrx19 in the presence of different substrates. Data presented are average values ± SD of *n* = 3 experiments. n.d. not detected.

Substrate	Peroxidase Activity
Ascorbic acid	n.d.
NADH	n.d.
Indole-3-acetic acid (IAA)	0.01 ± 0.00
Ferulic acid	0.53 ± 0.07
Coniferyl alcohol	1.46 ± 0.13
Sinapyl alcohol	0.08 ± 0.01

**Table 5:** Apparent *K*_M_, *K_cat_* and *K_cat_*/*K*_M_ values for coniferyl alcohol (CA) and sinapyl alcohol (SA) shown by PpaPrx19.

Substrate	*K*_M_ (μM)	*K*_cat_ (s^−1^)	*K*_cat_/*K*_M_ (μM^−1^ s^−1^)
CA	16.7	3940.0	235.8
SA	20.8	281.0	13.5

**Table 6:** List of proteins PpaPrx19 (PP1S306_37V6.1) has interactions with, based on STRING.

*P. patens* Protein ID	*A. thaliana* Homolog		Function	Score
PP1S84_209V6.1	CADG	AT1G72680.1	Cinnamyl alcohol dehydrogenase	0.650
PP1S126_185V6.1	CAD9	AT4G39330.1	Cinnamyl alcohol dehydrogenase	0.650
PP1S163_63V6.1	ELI3-2	AT4G37990.1	Cinnamyl alcohol dehydrogenase	0.650
PP1S56_71V6.1	OMT1	AT5G54160.1	O-methyltransferase	0.650
PP1S123_38V6.1		AT1G78780	PR protein	0.546
PP1S34_74V6.1		AT1G29850	DNA binding	0.546
PP1S141_102V6.3	MBF1A	AT2G42680.1	Multiprotein bridging factor	0.531
CHI		AT2G43590	Chitinase	0.521
PP1S96_94V6.1	MBF1B	AT3G58680.1	Multiprotein bridging factor	0.400
PP1S35_215V6.1	MBF1B	AT3G58680.1	Multiprotein bridging factor	0.400

## Data Availability

The data is contained within the article.

## Supplementary Materials

The following are available online at https://www.mdpi.com/article/10.3390/plants10071476/s1, Figure S1: Effect of different abiotic stresses on *PpaPrx19* gene expression, Figure S2: Graphical representation of the proteins PpaPrx9 (PP1S306_37V6.1) has interactions with. Table S1. List of coexpression patterns obtained for PpaPrx19 in Phytozome (https://phytozome.jgi.doe.gov/pz/portal.html), accessed on 8 July 2021.
