# Supplementary material for: Evolutionary Implications of a Peroxidase with High Affinity for Cinnamyl Alcohols from Physcomitrium patens, a Non-Vascular Plant
Source: Plants (Basel). 2021 Jul 19;10(7):1476. doi: 10.3390/plants10071476 (PMC8309402; doi:10.3390/plants10071476)
Supplement: Supplementary file 1 [file plants-10-01476-s001.zip › plants-1286984-supplementary.pdf]

Title: Evolutionary implications of a peroxidase with high affinity for cinnamyl alcohols from *Physcomitrium patens*, a non-vascular plant

Authors: Teresa Martínez-Cortés, Federico Pomar and Esther Novo-Uzal

Figure S1. Effect of abiotic stress on *PpaPrx19* gene expression. 18S was used as housekeeping gene. Values are mean ± SD (*n* = 3 independent experiments), asterisks indicate statistical differences (t-student, $p<0.05$) with control.

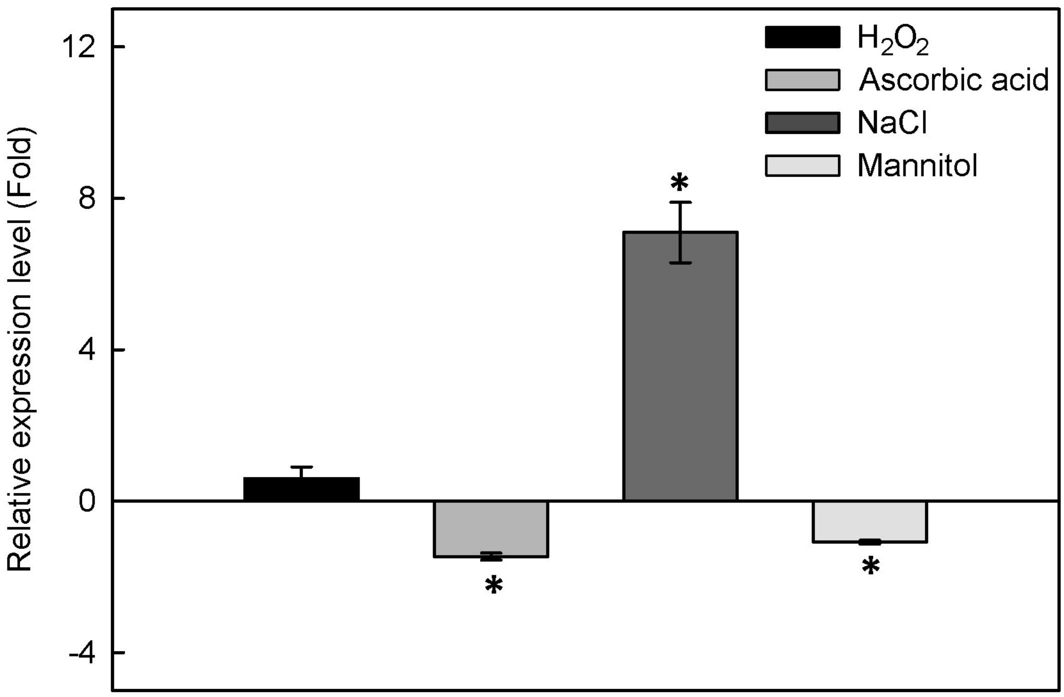

Figure S2. Graphical representation of the proteins PpaPrx9 (PP1S306_37V6.1) has interaction with, retrieved from STRING

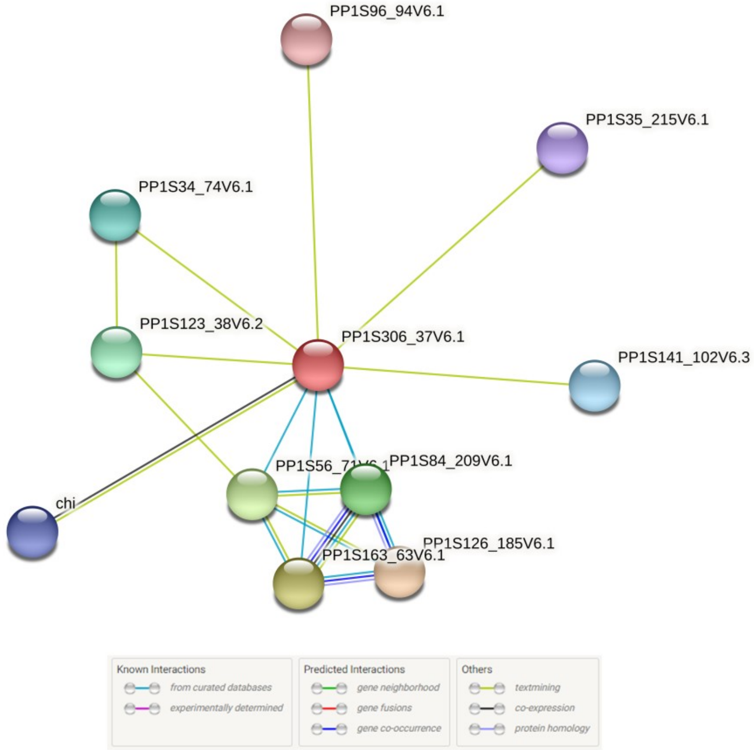

Table S1. List of coexpression pattern obtain for PpaPrx19 in Phytozome (https://phytozome.jgi.doe.gov/pz/portal.html)

P. patens ID	Protein	Correlation
Pp3c2_18040	Ancient ubiquitous protein 1	0.937
Pp3c7_5330	Leucine Rich Repeat (LRR_1)	0.921
Pp3c15_640	Auxin influx carrier (AUX1 LAX family)	0.883
Pp3c17_5130	beta-1,3-glucanase 1-related	0.877
Pp3c1_18680	Phenylalanine ammonia-lyase (PAL)	0.867
Pp3c8_5110	WRKY DNA -binding domain (WRKY)	0.860
Pp3c1_29970	Exostosin heparan sulfate glycosyltransferase-related	0.856
